# Alkali-Polymer Flooding in an Austrian Brownfield: From Laboratory to Field—Insights

**DOI:** 10.3390/polym16243607

**Published:** 2024-12-23

**Authors:** Muhammad Tahir, Rafael Hincapie, Torsten Clemens, Dominik Steineder, Amir Farzaneh, Silvan Mikulic

**Affiliations:** OMV Exploration & Production GmbH, 1020 Vienna, Austria

**Keywords:** emulsions, recovery factor, interfacial tension, alkali-polymer, heterogeneous, pilot project

## Abstract

We focus on optimizing oil displacement in brownfields using alkali polymers (AP) flooding. The goal is to enhance rock–fluid and fluid–fluid interactions to improve oil recovery. The evaluation includes detailed screening of AP mixtures to ensure cost-effectiveness and maximize chemical slug efficiency, using an AP pilot project in Austria as a case study. Key aspects of the study involve assessing fluid properties to select appropriate chemical concentrations. Important parameters include the stability of produced emulsions, interfacial tension (IFT) measurements, and rheological analyses. Rock–fluid interactions were examined through core flooding experiments on both low- and high-permeability core plugs to understand fluid dynamics in heterogeneous reservoirs. A novel part of the research involved simulating the in situ aging of the AP slug, which increases its anionicity over time. Two-phase core flooding with aged chemicals provided insights into the evolution of chemical effectiveness and interactions. We found that an alkali concentration of 7500 ppm was optimal for the AP slug, particularly in its interaction with dead oil with a high total acid number (TAN), leading to emulsions with microscopic instability. Single-phase core flooding showed that the AP slug from Vendor B outperformed that from Vendor A despite mechanical stability issues. However, the additional recovery factor (RF) for polymer A-based slugs was higher in both high- and low-permeability core plugs. The findings suggest that in situ aging of the AP slug could reduce costs and enhance injection performance.

## 1. Introduction

Polymer flooding has become a widely adopted technology for chemically enhanced oil recovery (cEOR) in mature oil fields, driven by a better understanding of recovery mechanisms and historical field application data [1,2,3,4]. However, polymer slugs often induce limited fluid–fluid interactions, leading to lower recovery factors compared to combined chemical EOR technologies [5,6,7,8]. To overcome these limitations, the combination of polymer slugs with other chemicals or co-solvents, such as alkali and surfactants, has been shown to significantly enhance oil recovery [9,10,11,12,13]. Nevertheless, the field application of combined chemical agents remains the same as polymer flooding applications [14]. Combination chemical agents, which involve multiple chemical reactions and intricate mechanisms, pose significant challenges for qualification processes in field applications. This is particularly due to the high costs associated with chemical agents [15].

Alkali polymers (AP) flooding has been demonstrated as an effective cEOR method for crude oil reservoirs with high total acid numbers (TAN). However, these findings have largely been confined to laboratory experiments focused on fluid–fluid and rock–fluid interactions [9,12,13,16]. In contrast, alkali–surfactant–polymer (ASP) and surfactant–polymer (SP) flooding have been successfully tested in the field and are well-documented in the literature [17,18,19]. The principal mechanism driving AP injection is the in situ generation of soap through the reaction of alkali with acidic polar compounds in crude oil [20,21]. However, it is imperative to note that a reduction in interfacial tension (IFT) via saponification is not always guaranteed [22]. Nonetheless, oils with TAN values exceeding 0.5 are considered suitable candidates for saponification. Furthermore, combining polymers with alkali allows for effective mobility control, thereby enhancing oil recovery [23,24,25]. AP systems produce unstable microemulsions, whereas polymer-free alkali systems form thermodynamically stable emulsions [26,27,28,29]. Microemulsion instability can trigger the mobilization of trapped oil ganglia by increasing local capillary numbers, thereby improving oil recovery [30]. Stable emulsions produced by polymer-free alkali are bigger in size and hard to break while flowing through porous media. Hence, stable emulsions can even block the pores owing to significantly higher viscosity and result in injectivity limitations. On the contrary, unstable microemulsions are smaller in size and result in phase separation (oil and aqueous phase) once mobilized. Hence, alkali produces soaps with reactive oil, lowering the IFT at the interface of the oil ganglia. Further oil ganglia are mobilized and produced owing to the sweep efficiency of the added polymer.

Despite the potential of multicomponent chemical agents, there are significant challenges limiting their field-scale application compared to polymer injection. One of the primary challenges in AP flooding is the deposition of silica scale in production wells, which requires preemptive mitigation measures [19,31,32,33]. Other notable challenges include the high cost of chemicals, which must be addressed to ensure economic viability [15,34,35,36], the handling of produced fluids (particularly emulsions), and the potential adsorption of chemicals onto reservoir rock surfaces during flow through porous media.

Several studies have emphasized that phase behavior screening in the laboratory is a critical step for determining the feasibility of AP systems, complemented by IFT measurements and displacement experiments in porous media (e.g., cores and micromodels) [11,12,13,27]. Arekhov et al. [37] and Schumi et al. [13] also investigated the types of alkalis used in the screening of optimal AP slugs under laboratory conditions. However, there remains a lack of clarity on whether variations in the average molecular weight of polymers from different suppliers and changes in the permeability of porous media affect the screening process. In our previous laboratory studies [11,12,13,27], we demonstrated that AP flooding is a cost-effective method capable of providing additional oil recovery in microscopic models and core flooding experiments. This additional oil recovery is largely attributed to the increase in local capillary numbers as alkali reacts with medium-viscosity crude oil. However, prior investigations were limited to a single polymer product and one core plug type. Therefore, the current study focuses on evaluating the performance of two different polymer products and core plugs with low and high permeabilities. This is to assess displacement efficiency and potential plugging issues. The rationale for using varying permeability is to better understand flow dynamics and injection performance. Clemens et al. [38] demonstrated that incremental oil recovery from polymer injection in the Matzen Field (8 TH reservoir) was primarily due to flow diversion into areas of lower permeability, rather than flow acceleration along primary pathways. For AP flooding, we anticipate a similar flow diversion effect, making it crucial to evaluate polymer–alkali interactions in rocks with varying permeabilities.

## 2. Approach

A series of experiments were designed and conducted to analyze fluid–fluid and rock–fluid interactions. The following procedures were employed in the experimental investigations:

- Phase behavior experiments and interfacial tension measurements were conducted to evaluate fluid–fluid interactions.
- Viscosity and volume of the produced emulsions were measured to determine the type of emulsion.
- Single-phase core flood experiments were performed to understand the chemical slug injectivity.
- The viscosity of core flood effluents was measured to clarify possible mechanical degradation.
- Two-phase core floods were used to study chemical slug efficiency and oil recovery.

## 3. Reservoir Data and Materials

### 3.1. Rock Properties

The feasibility study for evaluating the efficiency of the chemical slug (AP) targeted the 8 TH reservoir (Torton Horizon) in the Matzen field, located in the Schönkirchen area. This reservoir is a clastic, heterogeneous system with permeabilities ranging from 150 millidarcies (mD) to several darcies. The reservoir has an average thickness of 5 m and a porosity range of 28% to 30%. The formation brine salinity is 15.84 g/L, and the reservoir temperature is 49 °C.

For the study, Berea outcrop sandstone core plugs were used to investigate rock–fluid interactions. The high-permeability cores had an average brine permeability of approximately 300 mD, while the low-permeability cores ranged from 60 to 120 mD. Small core plugs (6 cm in length) were employed for single-phase core flooding experiments, whereas larger core plugs (30 cm in length) were used for two-phase flooding to mitigate capillary end effects. Scheurer [39] reported computed tomography results of high permeability sister core plugs to determine the presence of any inhomogeneities. Saleh [40,41] also reported that the pore walls were covered with feldspar or clay.

### 3.2. Fluid Properties

This experimental study utilized crude oil sampled from the S-85 production well. The oil was moderately degraded and capable of reacting with alkali to initiate saponification. According to SARA analysis of the dead oil sample, the composition consisted of 42% aromatic compounds, 39% saturates, 16% resins, and 3% asphaltene [42]. The total acid number (TAN) of the crude oil was 2.14 mg KOH/g, and the saponifiable acid content was 41 μmol/g based on a 7.7 g/L Na_2_CO_3_ solution. The dead oil had a viscosity of 56 mPa·s and a density of 0.891 g/cm^3^ at reservoir temperature. This dead oil was used for interfacial tension (IFT) measurements and phase behavior tests. For core flood experiments, however, cyclohexane was mixed with the crude oil to adjust the viscosity to a target value of 20 mPa·s at the reservoir temperature.

As presented in Table 1, two types of brine were used in this study. The first, 8 TH WTP, represents a simplified formation of brine containing calcium (Ca^2+^) and magnesium (Mg^2+^) ions. The second brine, referred to as soft 8 TH WTP, was a softened version of the first brine. The 8 TH WTP brine was used for the initial brine saturation of the core plugs, for permeability calculations, and during brine flooding sequences. In contrast, soft 8 TH WTP was used for alkali–polymer (AP) slugs.

To evaluate sweeping efficiency, we tested two high molecular weight hydrolyzed polyacrylamide (HPAM) polymer products from different suppliers. Both polymers were copolymers of acrylamide and acrylic acid, and neither was sulfonated. Polymer A, sourced from the first supplier, had an average molecular weight (Mw) of 22–25 million daltons (MDa), while polymer B, from the second supplier, had an average molecular weight of 18–22 MDa. The concentrations of both polymer products were optimized to achieve a final target viscosity of 24 mPa·s at 49 °C with a shear rate of 7.94 s^−1^.

**Figure 1:**
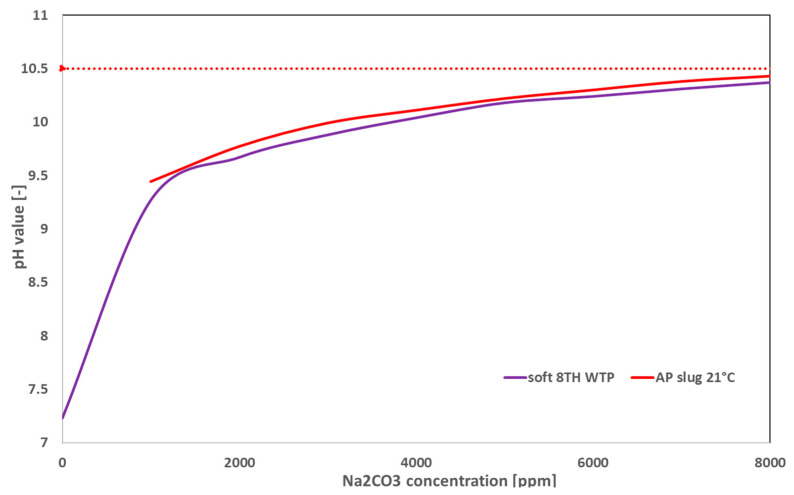
Impact of increasing alkali concentration on the increase in pH value.

Sodium carbonate (Na_2_CO_3_) was selected as the alkali for the preparation of AP solutions due to its cost-effectiveness and ability to prevent the risk of silica scaling in production wells, which can result from silicate dissolution [12]. For the two-phase core flooding experiments, aged AP slugs with the target viscosity were provided by both suppliers in specially designed cylinders. To establish baseline conditions, four two-phase core floods were conducted using unaged (in-house prepared) AP slugs. For the remainder of the experiments, AP slugs with the target viscosity were prepared in-house. A Na_2_CO_3_ concentration of 7500 ppm was selected to maintain the pH below 10.5 [9,13,43], as illustrated in Figure 1. Figure 1 shows the increase in pH with increasing alkali concentration. It is observed that the pH of the polymer solution (polymer B) is slightly higher than that of the base brine, likely due to the negative charges within the polymer’s molecular structure. A pH above 10.5 could increase the risk of silica scale formation; thus, the objective is to achieve a high pH without exceeding 10.5 value. Additionally, interfacial tension (IFT) results and phase behavior tests further validate the use of the target concentration of 7500 ppm of Na_2_CO_3_.

## 4. Methods

### 4.1. AP Slug Preparation and Rheology Measurements

#### 4.1.1. Two-Phase Core Flood

In two-phase core flooding, the AP solution provided by the supplier was aged in a glovebox using a stainless-steel cylinder. Nurmi et al. [12] reported an aging process that simulates the viscosity Increase in AP slug in reservoirs under reservoir conditions. Therefore, the aging method helps to determine the optimal slug concentration to achieve the target viscosity of 24 mPa.s (at 49 °C and 7.944 s^−1^), thus reducing costs. Aging reduces injection slug concentration and increases injection performance. Field aging under reservoir conditions improves slug viscosity and sweep efficiency. Polymer A reaches the target viscosity at 1300 ppm, while polymer B reaches the target viscosity at 1400 ppm. This is consistent with the molecular weight values. For confirmation, the viscosity of the delivered AP slug was measured using a Kinexus Pro+ rheometer prior to core flooding. For the base case of two-phase core flooding, the AP solutions were prepared in-house using the procedure described in the following step.

#### 4.1.2. For Single-Phase Core Flood

For both polymers, AP slugs were prepared in-house in soft 8 TH WTP brine with a target viscosity of 24 mPa.s at a temperature of 49 °C and a shear rate of 7.944 s^−1^. Stock solution was prepared with 5000 ppm concentration using an overhead mixer and was further diluted to the target concentration using a magnet-plate stirrer. Polymer A reaches 1500 ppm, while polymer B reaches 1850 ppm, with a viscosity of 24 mPa.s at reservoir temperature and an alkali concentration of 7.5 g/L. Scans of both polymers at various concentrations and temperatures using a Kinexus Pro+ rheometer showed a concentration decrease of 200 ppm for polymer A and a concentration decrease of 450 ppm for polymer B owing to the aging process.

### 4.2. Fluid–Fluid Interactions

#### 4.2.1. Interfacial Tension Measurements

IFT measurements were conducted using a spinning drop tensiometer. An oil sample from the 8 TH reservoir was introduced into an in-house prepared alkali–polymer solution. To minimize measurement errors, three repeat experiments were performed using the same set of solutions to calculate the average and standard deviation. Baseline measurements were established using soft 8 TH brine without polymer or alkali. Additionally, measurements were repeated utilizing an alkali-free polymer A solution as a base case. The Krüss spinning drop tensiometer was selected for its ability to measure the IFT between a heavy phase (e.g., alkali-polymer solution) and a lighter phase (e.g., oil). IFT measurements were performed on three AP solutions of polymer A with alkali concentrations of 5000 ppm, 6000 ppm, and 7500 ppm to investigate the IFT response of AP solutions at varying alkali concentrations. For polymer B, IFT measurements were conducted only at 7500 ppm alkali concentration.

#### 4.2.2. Phase Behavior Evaluations

To study the stability of the produced emulsions, three AP solutions were prepared for polymer A with a polymer concentration of 1850 ppm and varying alkali concentrations (5000 ppm, 6000 ppm, and 7500 ppm) to assess the effect of alkali content on emulsion stability. For polymer B, only one AP solution was prepared with a polymer concentration of 1500 ppm and an alkali concentration of 7500 ppm.

The phase behavior experiments involved sample preparation using 10 mL pipettes, which were sealed with a methane-oxygen flame. Each pipette was filled with 5 mL of the AP aqueous phase, followed by the addition of 5 mL of crude oil on top. The samples were thoroughly mixed using a rotary shaker at 50 rpm for 48 h. The pipettes were then stored in an oven at 49 °C, and the volumes of the different phases in each sample were monitored over time. To ensure reliability, multiple experiments were conducted with each solution. In addition to the pipette experiments, the emulsion stability was further evaluated using larger glass bottles (~200 mL) to measure the viscosity of the produced emulsions and to characterize the dead oil properties.

### 4.3. Rock–Fluid Interactions

#### 4.3.1. Single-Phase Core Flooding

**Figure 2:**
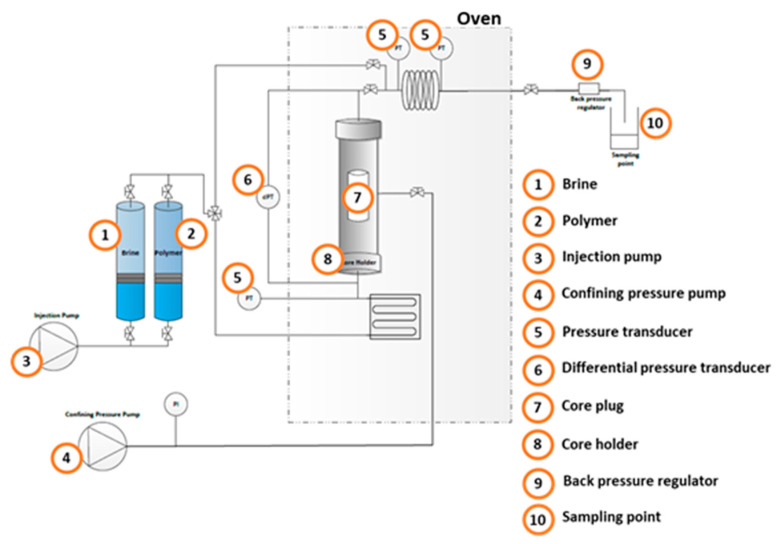
Experimental setup used for injectivity tests.

Injectivity Test: Single-phase core flood experiments were conducted using a custom-built setup, as illustrated in Figure 2. The apparatus was designed to flood Berea core samples with various aqueous solutions containing different types of alkalis and/or polymers. This allowed the observation of their effects on effective permeability.

Routine core analysis techniques were adapted for core initialization. The core was placed in the holder, and a radial confining pressure of 40 bar (g) along with a pore pressure of 5 bar (g) was applied. To remove air from the core plug, CO_2_ gas was continuously injected for 30 min. The cores were then saturated with synthetic formation brine at a pore pressure of 5 bar (g). The temperature within the heating cabinet was gradually increased to 49 °C, and brine permeability was measured. Following this, the samples were unloaded and weighed, and the pore volume was determined using Archimedes’ method.

The injectivity testing phase involved placing the core at a reservoir temperature of 49 °C, injecting hardened brine into the core, and measuring the pressure response. Brine permeability was evaluated at multiple injection rates to assess flow characteristics. In the second stage, chemical slugs (AP) were injected at a rate of 10 feet/day for 24 h. Injection rates were varied to collect effluents and assess any potential mechanical degradation of the polymer. In the final stage of the core flooding experiment, synthetic formation brine was re-injected, as detailed in Table 2.

To quantify the flow restriction imposed on the rock by adsorption of the aqueous phase, the resistance factor (RF) and the residual resistance factor (RRF) were calculated. These two parameters are determined using the following formulas:

(1) $$RF=\frac{\lambda_{brine}}{\lambda_{\mathrm{AP}\;solution}}$$

(2) $$\lambda =\frac{q\times L}{A\times \phi \times \Delta P}$$

(3) $$RRF=\frac{\lambda_{brine\;before\;\mathrm{AP}\;injection}}{\lambda_{brine\;after\;\mathrm{AP}\;injection}}$$

where the following apply:

Δ*P* = pressure drop along the core (mPa);

*ϕ* = porosity (−);

Q = injection rate (cm^3^/sec);

L = length of core plug (cm);

A = cross-sectional area of core plug (cm^2^).

Mechanical Degradation of AP Slug: Polymers are subject to mechanical degradation when flowing through porous media, leading to viscosity loss. Understanding the relationship between mechanical degradation and injection rate is crucial, particularly in conditions near the injection wellbore and deeper within the reservoir. Therefore, a key focus of this study was to evaluate the mechanical degradation of polymer solutions under these conditions. The mechanical degradation rate of the solution is calculated using the following formula:

(4) $$\mathrm{Mechanical}\;\mathrm{Degradation}\;\mathrm{Rate}\;\left(\%\right)=\left(\frac{\eta_{\mathrm{Fresh}\;\mathrm{solution}}-\eta_{\mathrm{Effluent}\;\mathrm{from}\;\mathrm{core}}}{\eta_{\mathrm{Fresh}\;\mathrm{solution}}}\right)*100$$

where η stands for the bulk shear viscosity of the AP solution at a shear rate of 7.944 s^−1^ and temperature of 49 °C.

To simulate mechanical degradation associated with severe injectivity issues, polymer injection was conducted at higher rates (10 ft/day). Conversely, lower injection rates (1 ft/day) were employed to replicate conditions deeper in the reservoir. Since alkali–polymer (AP) solutions were the focus of this study, it was necessary to evaluate their rheological properties. The viscosity of the AP samples at 49 °C was measured using a Kinexus Pro+ rheometer. Viscosity values were recorded across a range of shear rates from 1 s^−1^ to 20 s^−1^. These measurements are critical for understanding the behavior of AP solutions under various flow conditions, both near the wellbore and within the reservoir.

Pre- and post-XRD Analysis of Core Plugs: This task focused on identifying potential changes in rock mineralogy resulting from alkali–polymer interactions within porous media. Initially, selected core plugs from both rock types were analyzed using X-ray diffraction (XRD) to assess any mineral composition changes. If significant mineralogical changes were observed in the XRD analysis, further investigation using X-ray fluorescence (XRF) and scanning electron microscopy (SEM) was planned to broaden the scope of the study. For mineralogical comparisons, post-flush sister plugs were compared to fresh core plugs to detect any variations in mineral content resulting from the experimental processes.

#### 4.3.2. Two-Phase Core Flooding

Initialization of Core Plugs: Similar to the single-phase core flooding procedure, a brine saturation step and routine core analysis were performed. However, longer core plugs (1 ft in length and 3.8 cm in diameter) were used for two-phase core floods to mitigate capillary end effects and minimize potential dead volume errors. After measuring brine permeability, the core was saturated with dead crude oil to achieve an initial brine saturation of approximately 30%. Produced fluids were collected, and initial brine saturation was verified using the Dean–Stark method. Careful monitoring of the pressure differential was carried out to detect any potential plugging effects caused by the injected oil. The core plugs were then stored in an oven at 49 °C for 3–4 weeks to undergo the aging process. Following aging, the core plugs were reloaded into the experimental setup. The effective permeability to oil was re-measured to identify any changes or fluid redistribution within the core.

The sequence of events: All slugs were injected at an interstitial velocity of 1 foot/day. The following injection sequences were used:

1. Injection of synthetic formation brine (8 TH WTP) up to ~1.6 pore volumes.
2. Chemical slug (AP) injection up to 2 pore volumes.
3. Injection of approximately twice the pore volume of synthetic formation brine as post-flush.

After the flooding experiments, the core samples were unloaded, and the final saturation was confirmed using Dean–Stark measurements. Produced effluents were collected in fractions of approximately 4.5 mL. Fluid volumes were measured visually by assessing fluid levels in the collection tubes. This method was used to determine the volumes of the oleic phase, emulsion, and aqueous phases in the fractionated samples. The elapsed time and pressure differential were recorded throughout each experiment to monitor dynamic conditions. Table 3 summarizes the eight experiments performed for two-phase core flood to establish the comparison of chemical slug efficiencies.

## 5. Results and Discussion

### 5.1. Fluid–Fluid Interactions

#### 5.1.1. Interfacial Tension Experiments

The droplet’s shape significantly changed at the later stages of the experimental run; hence, it needed to increase rpm to keep the oil drop elongated. The data collected over 282 min showed the minimum values of IFT reached by each solution and the equilibrium IFT at which stability was reached. Due to the elongated droplet shape, the Vonnegut method was adopted for low IFT values lower than one mN/m.

**Figure 3:**
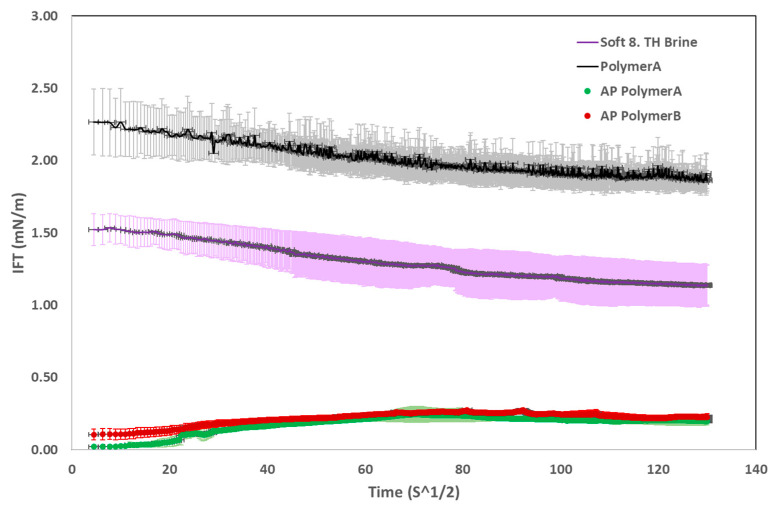
IFT measurements for AP slugs and two base cases without alkali (Polymer A and soft 8 TH WTP Brine).

On the other hand, the Young–Laplace method was adopted for higher IFT values due to their spherical shape (base cases). For the base case, two measurements with soft 8 TH WTP brine and a polymer solution, polymer A 1500 ppm, were also performed, as shown in Figure 3. As predicted, the AP solution resulted in much lower IFT values than the solutions without alkali (base cases). Interestingly, the first contact IFT value was around 10^−3^ mN/m for both AP solutions, but it is not shown in Figure 3. As oil drop formed at room temperature, the first contact IFT measured by the device was at room temperature. As the device took time to reach the target temperature of 49 °C, the measured IFT was increased to 10^−2^ mN/m during the heating process, which can be seen at the starting point in Figure 3.

Moreover, measurement error for base case measurements is higher than for the AP solutions. The reason for this could be that lower IFT values for AP solutions also result in lower standard deviation. As we can see, the polymer type has no impact on measured IFT; hence, both AP solutions resulted in the same response.

#### 5.1.2. Phase Behavior Evaluations and Emulsion Characterization

**Figure 4:**
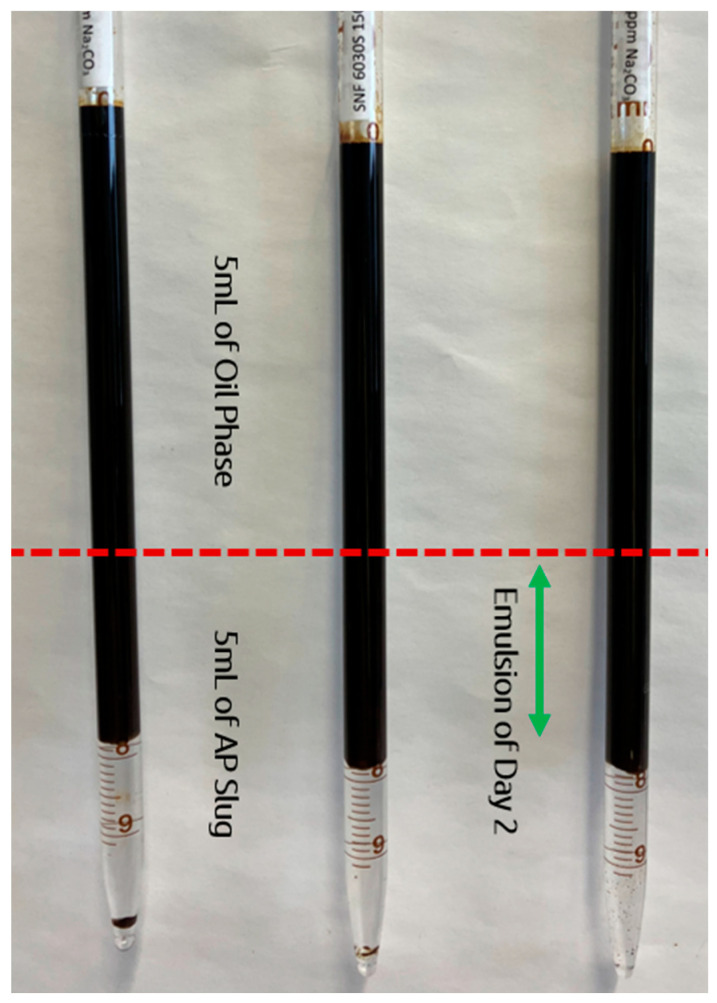
Produced emulsions on Day 2 for Polymer A-based AP slug. Three samples are for the same AP slug to consider the error bar in Figure 5.

**Figure 5:**
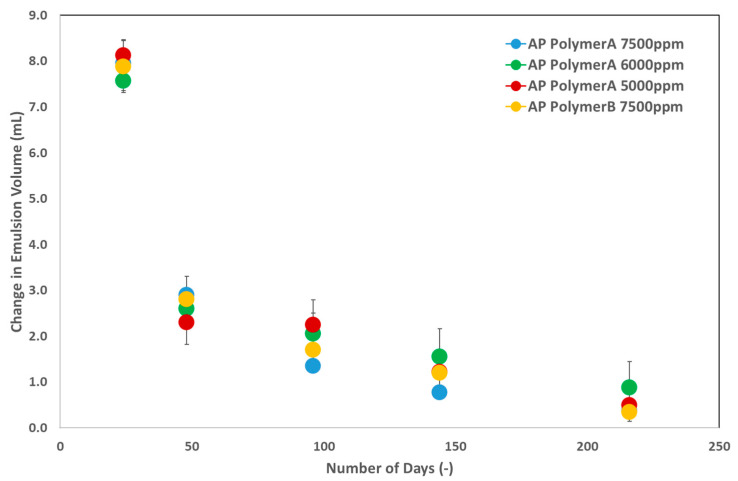
Change in produced emulsion volume versus time (days) measured in glass pipettes.

Phase behavior experiments were conducted to investigate the emulsification process and analyze the stability of the produced emulsions. Three chemical slugs of polymer A, each with a polymer concentration of 1500 ppm, were used to assess the emulsification process with an 8 TH oil sample, as shown in Figure 4. Figure 5 shows the alkali concentrations used: 5000 ppm, 6000 ppm, and 7500 ppm. Additionally, polymer B was prepared at a concentration of 1850 ppm and an alkali concentration of 7500 ppm. To reduce uncertainty, each sample was prepared three times, and the results are depicted in Figure 5, accompanied by an error bar.

As reported in our previous study [13], immediately after mixing, the alkali–polymer solution formed an emulsion phase alongside the main oleic and aqueous phases. However, the resulting emulsions were unstable, as shown in Figure 5. Over time, the emulsion volume decreased, leading to an increase in the oleic and aqueous phase volumes. This decrease in emulsion volume followed a similar trend across all AP solutions. Based on previous studies [9,10,12], a final alkali concentration of 7500 ppm was selected for further investigation. However, to evaluate the effects of lower alkali concentrations, 5000 ppm and 6000 ppm were also included in the phase behavior tests. Figure 5 shows that the emulsion volume and stability across the three alkali concentrations in the AP solution showed similar behavior. From an economic perspective, an AP solution with 5000 ppm of alkali is a favorable option. However, the primary challenge lies in alkali adsorption to rock surfaces. Therefore, the alkali concentration of 7500 ppm remains the focus for further studies on fluid–fluid interactions. Similar to the interfacial tension (IFT) measurements, the phase behavior tests conducted on pipettes concluded that the polymer type does not influence the volume of emulsion produced.

**Figure 6:**
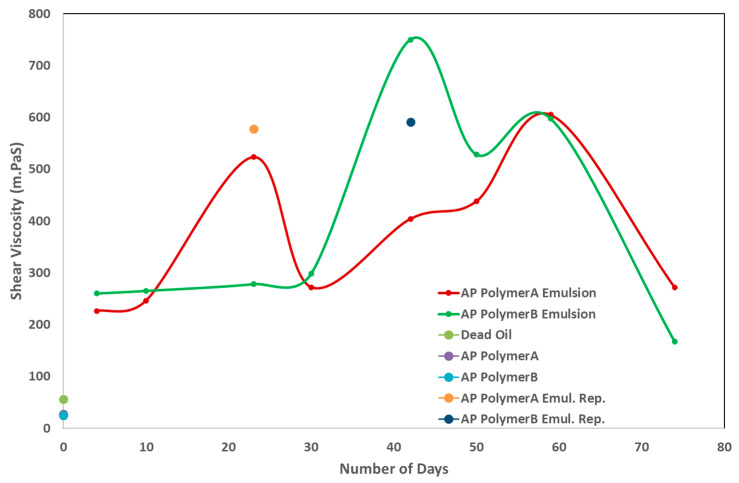
Change in emulsion viscosity measured over time (days) at a shear rate of 7.94 s^−1^ and temperature of 49 °C.

Emulsion volume changes were also observed in 200 mL glass bottles. However, the volume of the emulsions produced in the bottles decreased more slowly compared to those produced in the pipettes. One potential explanation is that the higher availability of alkali in the aqueous phase, combined with the carboxyl groups in the dead oil, resulted in maximum chemical interaction in the bottles, which have a larger geometry compared to the elongated pipettes with a smaller diameter. Furthermore, the higher length-to-diameter ratio of the pipettes allows for quicker separation of the aqueous and oil phases by gravity, unlike emulsions produced in bottles. As a result, phase separation in bottles occurred more slowly, and the emulsion viscosity did not follow a clear trend over several days. Initially, the prepared emulsion exhibited a much higher viscosity than both the original AP solution and the dead oil. This is illustrated in Figure 6. It can be observed that the emulsion viscosity increased significantly at first, followed by a gradual decrease to its initial value over time. Repeated viscosity measurements were performed, and the results are represented by a single point in Figure 6. Further, it can be seen that the produced emulsions were stable for about thirty days in the case of the polymer B product and can be seen as similar viscosity trend. However, polymer A-based emulsions showed instability, resulting in a viscosity increase and then decrease within 30 days. On the contrary, at a time step of more than 30 days, the viscosity of produced emulsions followed a similar trend for both products. Hence, the first thirty days are critical for comparing the instability of emulsions. Another factor to consider is the difference in molecular weight of the polymer products. Polymer A has a slightly higher molecular weight with a greater fraction of high molecular weight ends. Hence, emulsion stability can also be linked to polymer molecular weight but needs further testing. These changes in the emulsion volume and viscosity indicate that the emulsions produced were unstable and underwent phase separation over time. Additionally, the height-to-width ratio plays a crucial role in phase separation, especially considering the pore size distribution.

### 5.2. Rock–Fluid Interactions

#### 5.2.1. Single-Phase Core Floods

Injectivity Test: Table 4 lists the resistance factors (RF) and residual resistance factors (RRF) calculated from the core flooding tests. It can be observed that, for both low- and high-permeability core plugs, the RF value for polymer A is significantly greater than that of polymer B. This is a trend that was consistently replicated in repeated experiments. This suggests that polymer B may have better injectivity. However, to confirm this assumption, further investigation into the viscosity of the produced effluents is necessary. If polymer B shows a significantly higher viscosity loss compared to polymer A, the hypothesis of better injectivity would require further investigation. The higher molecular weight (Mw) of polymer A likely contributes to its higher RF, as larger polymer molecules may encounter greater resistance when penetrating pore throats.

Interestingly, polymer B demonstrated slightly lower RRF values in high-permeability core plugs compared to polymer A. In contrast, the low-permeability core plugs exhibited the opposite trend, with polymer A showing lower RRF values. As noted in Table 4, some experiments were performed three times rather than twice due to inconsistencies in the pressure response. This affected the calculation of RF and RRF. These inconsistencies were more pronounced in low-permeability core plugs than in high-permeability ones. One possible explanation is the pore size distribution within individual core plugs. Polymer solutions are non-Newtonian, experiencing shear-dominated flows that vary depending on the pore size distribution. Even minor changes in pore geometry can induce significant in situ rheological responses. Another notable observation is that polymer B exhibited more inconsistent behavior than polymer A in terms of pressure response. Additionally, polymer A consistently displayed significantly higher RF values in low-permeability core plugs.

Contrary to expectations, both polymers exhibited lower RRF values in low-permeability core plugs compared to high-permeability core plugs. However, the injection of the pore volumes (PV) required to achieve stable RRF values must be considered. For high-permeability core plugs, stable RRF values were obtained after injecting approximately 1–2 PV of brine. In contrast, for low-permeability core plugs, pressure differential stability was not reached, even after injecting 100 PV of brine. Consequently, the comparative injected pore volume and resulting RRF values for the high-permeability core plugs were lower than those of the low-permeability core plugs. However, the final stable RRF for low-permeability plugs was indeed lower.

**Figure 7:**
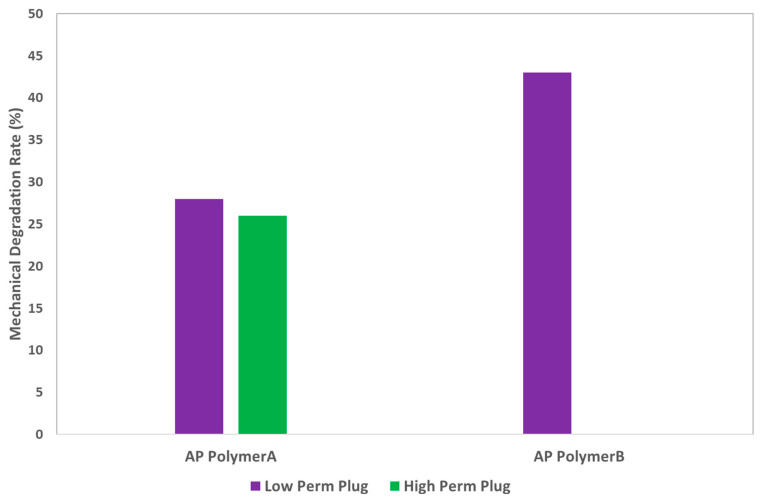
Mechanical degradation rate (%) of AP slugs injected at an injection velocity of 10 ft/day.

Mechanical Degradation Rate: Surprisingly, the effluent collected at the core outlet exhibited significant viscosity loss, as shown in Figure 7. Two polymer solutions (polymer A and polymer B) were injected into two types of core plugs (high permeability and low permeability) at an injection rate of 10 feet/day to simulate conditions near injection wells. Figure 7 illustrates that the viscosity loss for polymer B-based AP solutions was greater than for polymer A-based AP solutions. As expected, the mechanical degradation of the AP slug was more pronounced in low-permeability core plugs compared to high-permeability core plugs. This is likely due to the development of higher in situ shear rates in low permeability cores, resulting in greater viscosity losses. The higher viscosity loss observed for polymer B aligns with improved injectivity, as shown in Table 4. Therefore, the higher degradation and corresponding viscosity loss of polymer B is considered an indicator of enhanced injectivity. Further studies are currently underway to extend this work and explore this hypothesis in more detail.

**Figure 8:**
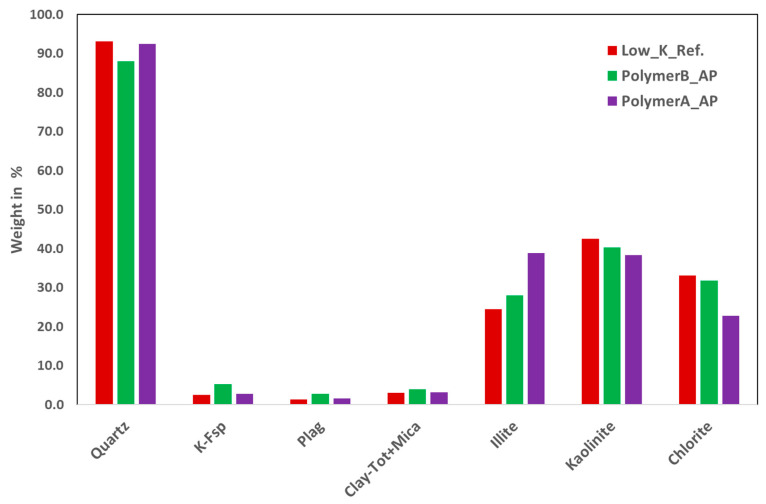
XRD analysis of low-permeability plugs for pre- and post-AP flooding conditions.

**Figure 9:**
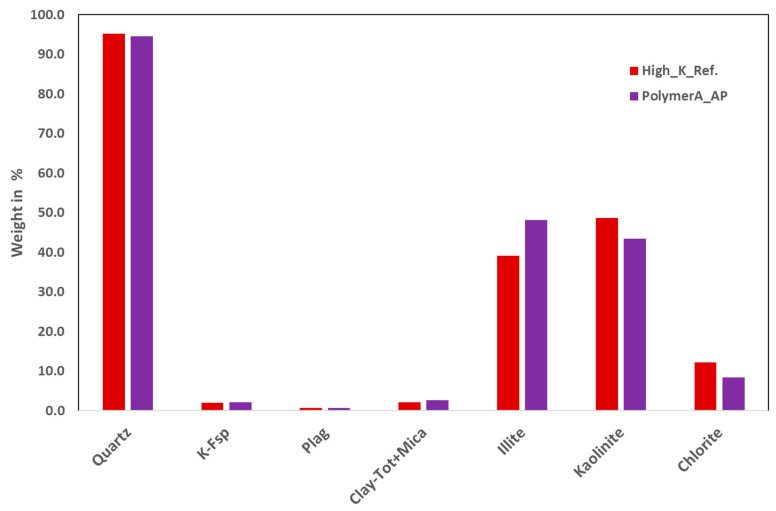
XRD analysis of high-permeability plugs for pre- and post-AP flooding conditions.

Pre- and Post-XRD Analysis of Core Plugs: Figure 8 and Figure 9 show the results of the XRD analysis of the two core plug types.

In both figures, the Ref. Legend represents a clean, unflushed core plug (before flushing), while the other core plugs were cleaned after AP slug injection (after flushing). As observed in the reference core plugs, there are mineralogical differences between the high- and low-permeability plugs. However, quartz remains consistent across both. The mineralogical differences between core plugs before and after flushing were not significant for a given sample. Experts believe that the observed variations may fall within the range of instrument measurement error. Therefore, based on the XRD results, it can be concluded that the alkali does not induce significant mineralogical changes in the porous media. As a result, further evaluation using XRF or SEM techniques is not deemed necessary.

#### 5.2.2. Two-Phase Core Floods

**Figure 10:**
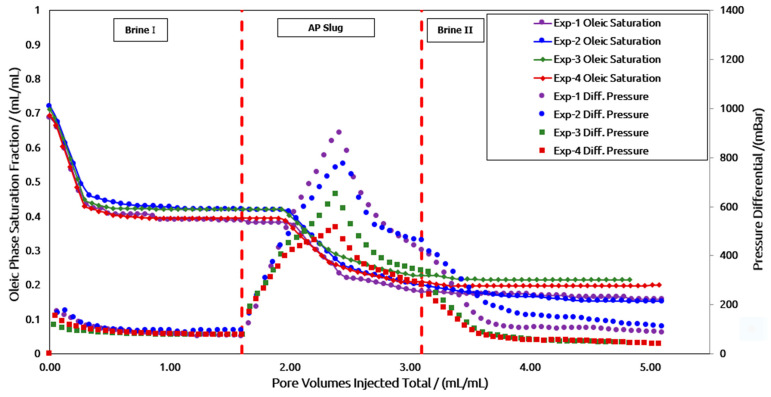
Two-phase core flood in high-permeability plugs using AP slugs from two vendors (aged and un-aged slugs).

As summarized in Table 3, four core flooding experiments were conducted on high-permeability core plugs, and another four were conducted on low-permeability core plugs using two different polymer products (polymer A and polymer B). Figure 10 presents the residual oil saturation curve along with the corresponding differential pressure changes for the high permeability core plug.

High-Perm Core Plugs: As shown in Figure 10, Experiment 1 represents the base case with unaged AP slugs, while Experiment 2 represents aged AP slugs made from polymer A. The trends in decreasing residual oil saturation and increasing pressure differential are similar in both experiments. Since the target viscosity of both AP slugs is 24 mPa.s, the pressure response trends are nearly identical. However, the aged AP slug demonstrates improved injectivity, with a lower peak pressure. Similarly, the decrease in oil saturation follows the same pattern, and the final oil recovery is almost the same, as shown in Table 5. Therefore, it can be concluded that the in situ aging process of the AP slug with polymer A can reduce the polymer concentration by approximately 200 ppm while maintaining the same oil recovery factor. This is attributed to the interaction between the alkali and the increased polymer anionicity, as suggested by previous research [9,12]. The aging process defines the maturation of the AP slug over time under anaerobic conditions. As a result of the aging process, the viscosity of the AP slug increases due to the development of repulsive forces in the aqueous phase as polymers have a negative surface charge, and the added amount of alkali provides more negative charge in the aqueous phase to increase the anionicity. Hence, the repulsive forces cause polymer chains to stretch out, as is seen as the viscosity gain for the AP slug. However, some concentration of alkali can be consumed to increase the AP viscosity, and its impact on lowering the IFT can be sacrificed. In the laboratory, the aging process is completed in a glovebox to establish an oxygen-free environment. However, in situ aging defines the maturation process inside the reservoir while flowing through porous media. The benefit of in situ aging is better injectivity of AP slug due to lower viscosity and then improved sweep efficiency once viscosity is increased due to the in situ aging process.

The same evaluation was conducted on polymer B. In Experiment 3, unaged AP slugs were injected, while Experiment 4 involved aged AP slugs. The pressure trend and reduction in oil saturation were similar, reflecting the comparable target viscosities of both slugs. As with polymer A, the peak pressure drop for the aged AP slug of polymer B was slightly lower than for the unaged slug, indicating improved injectivity. Table 5 shows that the final recovery factors (RFs) of Experiments 3 and 4 are comparable. Consequently, the aging process allows for a reduction of 450 ppm in the polymer B concentration while maintaining the same recovery factor.

Furthermore, a comparison of the two polymer products shows that the pressure drop for polymer A was approximately 250 mBar (30%) higher than for polymer B in both aged and unaged slugs, indicating that polymer B-based AP slugs had better injectivity. This observation is consistent with the single-phase core flood injection results presented in Table 4.

However, the final recovery factor of polymer B-based AP slugs was 7–8% lower than that of polymer A-based AP slugs. One possible reason for this is the higher molecular weight of polymer A, which provides better viscoelastic properties in porous media. The slightly higher pressure drop (ΔP) for polymer A suggests a more effective viscoelastic response, which researchers report contributes to significant additional oil recovery [44,45,46,47,48].

Looking at the additional recovery factor (RF) for each AP slug in Table 5, the performance differences between the products are within 3–4%. Therefore, careful selection of the appropriate AP slug based on the polymer type is required. Polymer B exhibited better injectivity (30% improvement), while polymer A demonstrated an additional 7% increase in oil recovery. Notably, the pressure drop for aged AP slugs was lower than for unaged slugs in both products. In conclusion, the in situ aging process improves the injectivity of chemical slugs by enabling the use of lower polymer concentrations during injection while reducing the pressure drop once the aging process is established in the reservoir.

Low-Perm Core Plugs: Figure 10 illustrates the reduction in oil saturation and the corresponding differential pressure response for a low-permeability core plug. This is achieved following the same AP injection sequence, as shown in Figure 10. However, under low-permeability conditions, comparing aged and unaged slugs is more challenging. A likely reason for this is the variation in permeability values, as detailed in Table 5. Significant changes in permeability can lead to inhomogeneities in the porous medium (such as the pore size distribution) and potentially different fluid distributions due to local capillary forces. As a result, the additional oil recovery factor varies for each slug, though the final oil recovery remains within a 4% difference. A comparison of the recovery factor (RF) values in Table 5 shows that high-permeability core plugs exhibit higher oil recovery during brine flooding compared to low-permeability core plugs. However, after AP flooding, oil recovery from low-permeability core plugs was considerably higher. This could be attributed to the higher remaining oil saturation in the low-permeability core plug after brine flooding. This allows for a more substantial interaction between the remaining oil and the injected AP slug.

**Figure 11:**
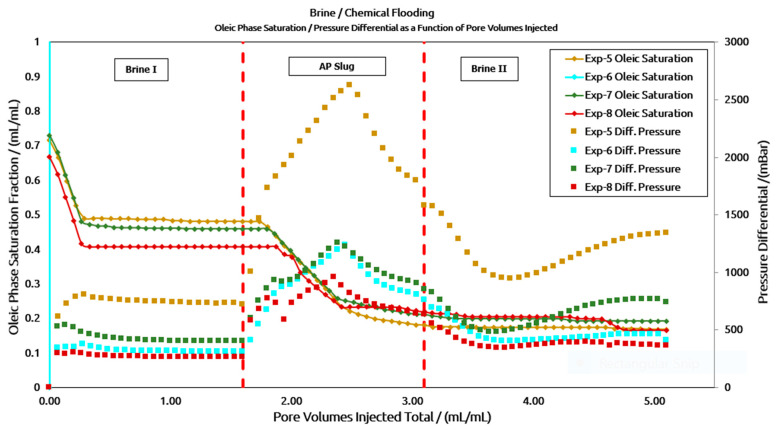
Two-phase core flood in low-permeability plugs using AP slugs from two vendors (aged and un-aged slugs).

**Figure 12:**
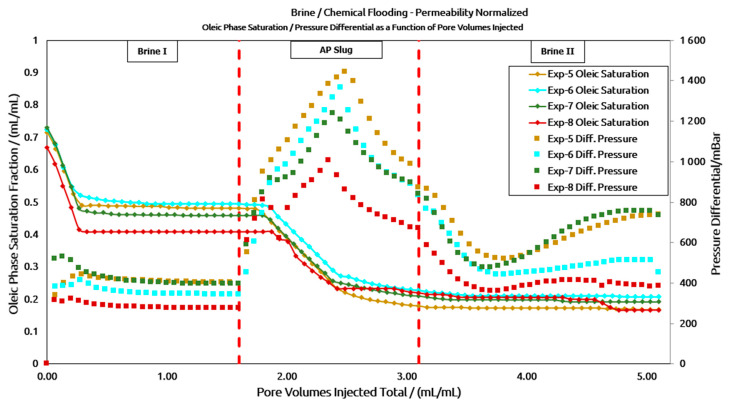
Results of low-permeability core floods with normalized pressure differential data.

In the differential pressure response shown in Figure 11, a higher pressure drop (ΔP) was anticipated for Experiment 5 due to the lower permeability value. The ΔP plots for the low-permeability cores were normalized for clarity, as illustrated in Figure 12. It can be observed that the ΔP response follows a similar trend to that of the high-permeability core plug. The peak ΔP for the polymer A-based AP slug is higher than that of the polymer B-based AP slug, both in the unaged slug (200 mBar) and the aged slug (360 mBar). Additionally, the peak ΔP of the unaged slug is slightly higher than that of the aged slug—80 mBar for polymer A and 235 mBar for polymer B. Another notable observation is the peak ΔP during post-brine flooding in the low-permeability core plugs, as seen in Figure 11 and Figure 12. As expected, the pressure response initially decreased but then unexpectedly increased after approximately 0.7–0.8 pore volumes (PV) of brine injection, corresponding to brine breakthrough. One potential explanation, as reported by the supplier, could be the chemical interaction between the alkali (CO_3_^2−^) in the AP solution and the divalent ions (Ca^2^⁺/Mg^2^⁺) in the chasing brine. Another contributing factor may be the high concentration of high molecular weight polymers combined with the low permeability of the core plugs. However, further investigation is required to determine the exact causes of this pressure increase.

### 5.3. AP Field Pilot Project

**Figure 13:**
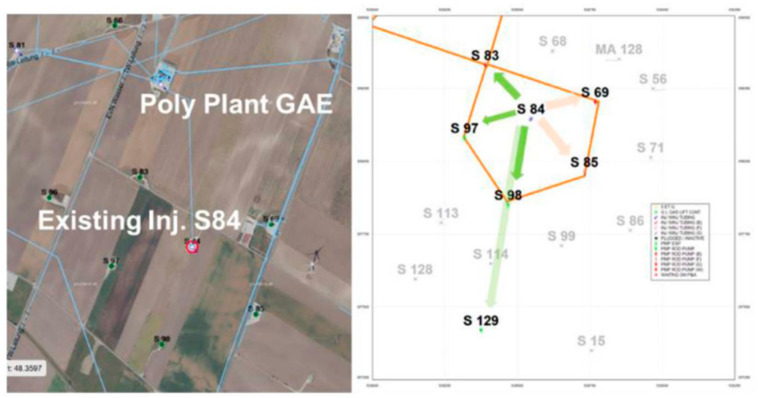
Injector-producer well pattern selected for AP pilot project [49].

Following extensive laboratory testing conducted in previous years [9,10,12], the decision was made to proceed with a field pilot project. A brief note outlining the risks and challenges associated with pilot testing was recently published [49]. The pilot study was designed around one injection well (S-84), surrounded by five production wells (S69, S83, S85, S97, and S98), as shown in Figure 13. All wells are vertical, with distances of 240–290 m between the injection well and the production wells. They are equipped with sucker rod pumps for production. The target reservoir for this pilot study is the 8. TH reservoir, located in the Matzen field region of Schönkirchen. This reservoir comprises shallow marine sandstone with a formation thickness of up to 20 m. It has a porosity range of 20–30% and an average permeability of 500 mD. This well pattern was selected due to its extensive characterization in previous experiments, tracer studies, and several enhanced oil recovery (EOR) applications. Given that most of the oil is produced from this pattern, the trial aims to validate the alkali–polymer (AP) application technology and address the challenges posed by produced fluids. Since additional oil recovery was not the primary goal of this study, polymer B, known for its superior injectivity, was chosen for the field test to minimize injectivity-related limitations. To mitigate potential polymer degradation at the injection well and pump, the injection recipe for the AP Slug consists of 1500 ppm polymer B and 7500 ppm alkali. The AP Slug flooding began in 2023 and is expected to continue until Q2 2025. Breakthrough of the AP Slug is anticipated in the coming months.

### 5.4. Laboratory and Field Observations Relation—Overall Observations

A thorough assessment of fluid–rock interactions is critical for optimizing alkali–polymer (AP) flooding in brownfields and essential for enhancing AP flooding efficiency. This study emphasizes the importance of tailoring AP formulations to achieve cost-effectiveness and maximize overall impact. Further testing on actual field rock samples is recommended to optimize AP flooding for brownfield applications. The following general observations were made:

- Although laboratory procedures for mixing and preparing alkali–polymer (AP) solutions differ from pilot-scale practices, the relationship between concentration and viscosity translates well, preventing polymer degradation at the injection site. Target viscosities for the AP slugs were employed in the pilot to ensure consistent concentrations.
- Wellhead pressure monitoring in the injection well showed no sustained decline in injection performance since the initial chemical injection in Q2 2023, except for temporary operational limitations.
- A significant volume of diluted brine (soft 8. TH WTP) was injected on-site but was not evaluated in the laboratory. This currently limits direct comparisons between field observations and laboratory results.
- A one-month injection test (April–May 2024) using a polymer A-based AP solution was also conducted to evaluate its full feasibility, as it offers similar injection characteristics to the polymer B-based AP slug and may positively affect project economics.
- AP injections will continue until a breakthrough is achieved, which is expected in Q4 2024/Q1 2025.

Several field observations can be categorized as follows:

Injector wellhead pressure: Based on geomechanical studies conducted on two core samples from the 8 TH reservoir, the wellhead pressure (WHP) was initially limited to a maximum value of 55 bar. This corresponds to a bottom hole pressure (BHP) of 180 bar, approximately 10% lower than the fracture pressure derived from core tests. As large-scale injection increased the pore pressure around the well, the corresponding fracture pressure also rose. Consequently, the maximum wellhead pressure was increased to 60 bar.

Reservoir pressure: With the exception of the AP project, where the injector was equipped with borehole pressure gauges to measure both borehole and reservoir pressure, no other wells were fitted with pressure gauges. Over the past year, most wells have been shut down for various reasons, including AP stage upgrades, well integrity issues, and pumping problems. During these shut-ins, the static fluid level was measured to estimate reservoir pressure, which averaged around 80–85 bars. This approach is common in the Matzen field, given the absence of downhole gauges in production wells.

**Figure 14:**
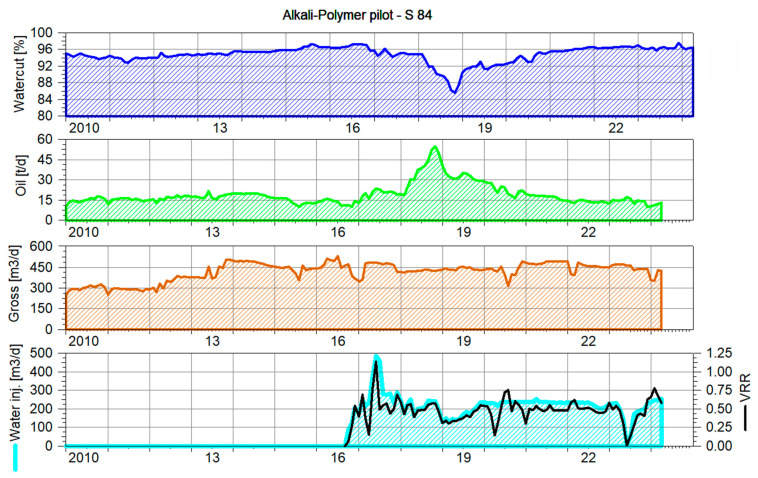
History production and Voidage Replacement Ratio (VRR) for the AP pattern area.

Production rates: The Voidage Replacement Ratio (VRR) for this pilot pattern stabilized at approximately 0.5 over the last three years, which is below the optimal value for maintaining adequate pressure support due to the lack of significant aquifer drives. The production history and VRR for the AP test area are shown in Figure 14. This lower VRR results in a dynamic fluid level drop, though there is no immediate risk of pump-off.

Production from well S83 has been intentionally reduced (in gross production) to redistribute AP flow paths in the subsurface and direct a larger portion of the AP slug toward the S97 and S98 production wells, which show better oil production potential. While S83 achieved the first polymer and tracer breakthroughs, its lower oil saturation zone resulted in a less significant production increase in terms of volumes produced. The main production wells are equipped with real-time dynamometer systems to monitor pump efficiency and load during scale build-up or sudden pump-off events. These systems ensure accurate monitoring of the pump string’s up-and-down movement, safeguarding against performance issues during AP slug flooding.

## 6. Conclusions

This study highlights the importance of a thorough investigation into fluid–fluid and rock–fluid interactions to enhance oil displacement efficiency by alkali–polymer (AP) flooding in brownfields. Detailed screening of AP mixtures proved essential for cost-effectiveness and maximizing chemical slug efficiency. The evaluation of these interactions led to the following key conclusions:

- The alkali concentration scan revealed that 5000–7500 ppm of Na_2_CO_3_ is required to maintain the target pH range of 10–10.5. However, the presence of polymers causes a slight increase in pH due to the negatively charged polymer molecules.
- IFT measurements showed that alkali significantly reduced IFT values compared to the base case. The first contact IFT was measured at approximately 10^−3^ mN/m, while equilibrium IFT values stabilized at around 10^−2^ mN/m. Additionally, the polymer type and molecular weight distribution had no significant influence on the IFT response.
- Phase behavior tests (emulsion volume and viscosity) indicated that the produced emulsions were slightly unstable, leading to a reduction in emulsion volume and viscosity changes over time. The sample container width-to-height ratio also strongly influenced emulsion stability. At alkali concentrations of 5000–7500 ppm, both polymer products produced similar emulsion volumes.
- Polymer B-based AP slugs exhibited better injectivity in both high- and low-permeability core plugs. However, mechanical degradation rates indicated that effluents from polymer B-based core plugs experienced greater viscosity loss compared to polymer A. Interestingly, low-permeability core plugs demonstrated lower residual resistance factor (RRF) values than high-permeability core plugs. The pore volume (PV) injection to achieve stable RRF must be considered. For high-permeability cores, 1–2 PV of brine resulted in stable RRF values. In low-permeability cores, pressure stability was not reached even after 100 PV of brine injection.
- XRD analysis of the core plugs, pre- and post-injection, showed that alkali did not chemically react with the rock material, suggesting no mineralogical changes. However, this may not hold true for real field rock samples, and further testing is recommended.
- While polymer B demonstrated better injectivity in two-phase core flooding, polymer A-based AP slugs contributed to higher oil recovery in both high- and low-permeability core plugs. Polymer B was selected for the AP pilot application, which will run until Q1 2025.
- The in situ aging process reduces polymer usage, improving project economics for field-scale applications. The aging process also enhances AP slug injectivity by allowing for lower polymer concentrations (200 ppm reduction for polymer A and 450 ppm for polymer B).

## Figures and Tables

**Table 1:** Composition of both brines used for this study.

Salt	8 TH WTP (g/L)	Soft 8 TH WTP (g/L)
NaCl	22.53	22.62
KCl	0.16	0.16
MgCl_2_ ∗ 6H_2_O	0.72	-
CaCl_2_ ∗ 2H_2_O	0.94	-
NaHCO_3_	-	1.52
TDS	24.35	24.3

**Table 2:** Injectivity-focused single-phase core flooding experiments.

Res/Temp	Rock Type	Rock Condition	Sat. Brine (Swi)	Injection Sequence
8 TH/49 °C	Berea (Kb = ~200 mD) Outcrop	Cleaned via Soxhlet Extraction	8 TH WTP	8. TH Brine AP 7500 ppm Na_2_CO_3_ + 1500 ppm Polymer A 8. TH Brine
				8. TH Brine AP 7500 ppm Na_2_CO_3_ + 1850 ppm Polymer B 8. TH Brine
	Berea (Kb = ~100 mD) Outcrop			TH Brine AP 7500 ppm Na_2_CO_3_ + 1500 ppm Polymer A 8. TH Brine
				TH Brine AP 7500 ppm Na_2_CO_3_ + 1850 ppm Polymer B 8. TH Brine

**Table 3:** Summary of AP slugs used for two-phase core floods. AP slugs are prepared in soft 8 TH WTP brine and with an added alkali of 7500 ppm (Na_2_CO_3_).

Exp. #	Polymer Concentration	Slug Type	Core Type	Comment
1	1500 ppm Polymer A	Unaged	High Perm	Base Case
2	1300 ppm Polymer A	Aged		Cost-effective case
3	1850 ppm Polymer B	Unaged		Base Case
4	1400 ppm Polymer B	Aged		Cost-effective case
5	1500 ppm Polymer A	Unaged	Low Perm	Base Case
6	1300 ppm Polymer A	Aged		Cost-effective case
7	1850 ppm Polymer B	Unaged		Base Case
8	1400 ppm Polymer B	Aged		Cost-effective case

**Table 4:** RF and RRF values for single-phase polymer floods at the injection velocity of 10 ft/day using low-perm and high-perm core plugs.

Exp.	Core Type	Chemical Slug 1	*RF* @ 10 ft/Day	RRF @ 10 ft/Day
1	High-Perm	Polymer A 1500 ppm + 7500 ppm Alkali	253	27
2			260	22
3		Polymer B 1850 ppm + 7500 ppm Alkali	95	18
4			105	17
5	Low-Perm	Polymer A 1500 ppm + 7500 ppm Alkali	186	3
6			178	5
7		Polymer B 1850 ppm + 7500 ppm Alkali	63	10
8			48	5
9			51	12

**Table 5:** Summary of core plugs' petrophysical properties and results from two-phase core flood experiments.

Parameter	Units	Exp. 1	Exp. 2	Exp. 3	Exp. 4	Exp. 5	Exp. 6	Exp. 7	Exp. 8
C_Alkali_ in AP	g/L	7.5							
C_Polymer_ in AP	g/L	1.5	1.3	1.85	1.4	1.5	1.3	1.85	1.4
Polymer Vendor	g/L	A		B		A		B	
AP Slug Type	-	Un-aged	Aged	Un-aged	Aged	Un-aged	Aged	Un-aged	Aged
Length	cm	30							
Diameter	cm	3.8	3.8	3.8	3.8	3.8	3.8	3.7	3.8
Bulk Vol.	cm^3^	343	341	344	341	330	331	330	331
Pore Vol.	cm^3^	70	66	69	70	59	64	65	65
Porosity	%	21	19	22	21	18	19	20	20
Brine Perm.	mD	350	267	369	356	66	132	118	126
Init. Oil Sat.	%	69	72	71	70	72	72	73	67
RF Brine	%	43	41	41	43	33	32	37	39
Additional RF AP	%	30	31	27	27	42	37	34	28
Additional RF Brine	%	3	7	2	1	2	2	3	8
Total RF	%	77	79	70	71	77	71	74	75

## Data Availability

The raw data supporting the conclusions of this article will be made available by the authors upon request.
